# Multi-Scale Numerical Simulation of Fatigue Crack Propagation Mechanisms in the Heat-Affected Zone of AH36 Steel Welds

**DOI:** 10.3390/ma19091680

**Published:** 2026-04-22

**Authors:** Chaoming Shen, Yuxiao Fu, Wei Zhao, Jianhua Yang

**Affiliations:** 1 School of Naval Architecture & Ocean Engineering, Jiangsu University of Science and Technology, Zhenjiang 212100, China; shencm@just.edu.cn (C.S.); 13984598425@163.com (Y.F.); hitchar@just.edu.cn (W.Z.); 2 Jiaxing Special Equipment Inspection and Testing Institute, Jiaxing 314000, China

**Keywords:** AH36 steel, weld heat-affected zone, fatigue crack propagation, multi-scale simulation, coupled molecular dynamics–finite element method (MD-FEM)

## Abstract

This study conducts multi-scale numerical simulations spanning atomic to macroscopic scales (i.e., from nanometer to millimeter scale) to investigate the fatigue crack propagation behavior in the welded heat-affected zone (HAZ) of AH36 shipbuilding steel. A coupled molecular dynamics–finite element method (MD-FEM) was employed to establish a multi-scale model. Through the transfer of boundary displacements, equivalent mapping of crack morphology, and crack-tip tracking, an iterative multi-scale simulation of 600 tension–tension fatigue cycles was achieved. The results indicate that the crack propagation rate is significantly influenced by crack tip morphology (blunting/sharpening) and growth direction. Notably, the peak strain at the boundary is not the sole determining factor. Periodic blunting of the crack tip occurs during cyclic loading, accompanied by a decrease in the propagation rate. Additionally, the stress field near the crack tip induces microscopic defects such as voids in the nearby area, affecting the crack propagation. This study, based on multi-scale analysis, reveals the microscopic mechanism and evolution law of fatigue crack propagation in the heat-affected zone of AH36 steel welds.

## 1. Introduction

Marine engineering structures of ships are mainly composed of welded shipbuilding steel and are subject to wave impact and alternating loads for a long time. Therefore, it is crucial to study the fatigue damage mechanism of their welded joints.

The heat-affected zone of the weld, under the effect of thermal cycling, shows a non-uniform continuous distribution of microstructure and properties, often hardening, embrittlement or softening, accompanied by microscopic defects, making it a weak link in the weld joint [1,2]. Under the effect of the working load, this area is prone to initiate microscopic defects and expand into cracks, so the study of welding fatigue should focus on this area [3].

Microstructural evolution of materials is a critical factor governing crack initiation and propagation. Chawla et al. [4] experimentally indicated that cracks tend to nucleate at stress-concentrated regions including surface depressions, microvoids, secondary phases and grain boundaries. Kou et al. [5] observed via in situ tensile tests and transmission electron microscopy (TEM) that dynamic twins at crack tips exhibit a dual effect on crack propagation: twin boundaries are susceptible to stress concentration and thus induce microcracks, whereas twin formation can dissipate energy at the crack tip and raise the crack propagation threshold. Kachanov’s group [6] developed a new crack evolution model based on continuum damage mechanics. In this theoretical model, metallic materials first undergo internal micro-damage (e.g., microvoids and microcracks) under fatigue loading; macroscopic fracture occurs once the damage accumulates to a critical level. The Brown–Embury theory [7] revealed that microvoids form along 45° shear bands and coalesce with each other under applied stress. The Thomason model [8] proposed that shear stress triggers the nucleation of microvoids, which continuously emit dislocations, and the dislocation motion promotes the growth and expansion of microvoids.

In recent years, in situ techniques have advanced rapidly, enabling direct observation of microvoid formation by numerous researchers. Weck et al. [9] captured the full sequence of microvoid nucleation, coalescence and expansion. Baik [10] investigated the crack propagation behavior of high-manganese steel using in situ TEM and found that twins with regular geometric features appear at a certain distance ahead of the crack tip, and microvoid coalescence occurs as the crack approaches the twins. Mendoza et al. [11] observed the nucleation and growth of microvoids within shear bands near crack tips in titanium alloys.

In summary, as typical stress concentration sites, microvoids readily promote crack initiation. Driven by stress fields and dislocation motion, microvoids undergo continuous nucleation, aggregation, growth and coalescence. This process serves as a key microscale mechanism for fatigue damage accumulation, crack propagation and eventual macroscopic fracture in metallic materials.

AH36 steel, which has the advantages of high strength and toughness, easy weldability and corrosion resistance, is widely used in deep-sea engineering, green ships, polar navigation and other fields [12]. Liang et al. [13] conducted crack growth tests on AH36 steel under constant-amplitude and variable-amplitude tensile cyclic loading. The results show that AH36 steel is sensitive to the stress ratio effect under constant-amplitude tensile cyclic loading: the crack growth rate decreases remarkably with an increase in stress ratio and a decrease in stress amplitude. Meanwhile, the introduction of a single tensile overload into the tensile cyclic loading produces a pronounced overload retardation effect. With a larger overload ratio, the crack retardation growth rate becomes lower, the retardation zone becomes longer, the retardation effect becomes more significant, and the fatigue life is correspondingly prolonged.

**Figure 1:**
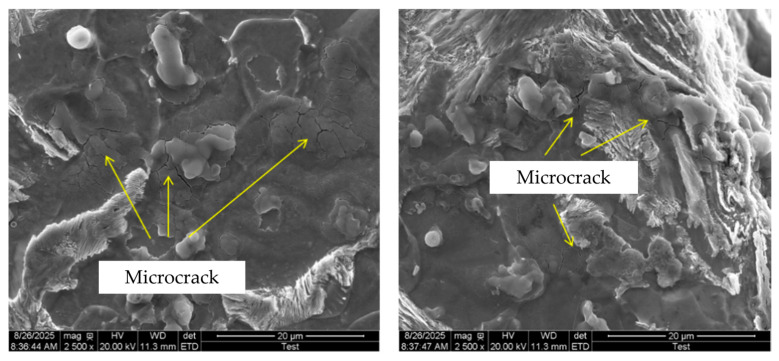
SEM micrograph of the heat-affected zone (HAZ) in an AH36 welded specimen after fatigue testing.

Fatigue tests were performed on welded AH36 steel specimens, and scanning electron microscopy (SEM) observations of the welded heat-affected zone (HAZ) revealed the presence of multiple microcrack defects in this region (see Figure 1). However, current research on the fatigue damage mechanism of the welded HAZ in AH36 steel remains insufficient, and relevant studies carry important academic value. Therefore, this paper selects AH36 steel to conduct multi-scale numerical simulations of fatigue crack propagation in the welded HAZ.

Material failure is a systematic problem involving coupling multiple spatiotemporal scales [14]. The multi-scale simulation method can couple different scales of analysis to reveal the damage mechanism more comprehensively. The macroscopic properties of metals are determined by their microscopic atomic structure, microstructure and cross-scale deformation mechanism, and cross-scale analysis helps to gain a deeper understanding of their mechanical behavior [15].

There are many classification methods for multi-scale analysis. Rudd [16] divided multi-scale research methods into serial and parallel based on whether different scales are coupled with each other. In the serial method, large-scale parameters are obtained through small scales, but the sets of variables at large and small scales are not coupled. The parallel method is mainly applicable to strong coupling of variables between scales. Computations at each scale need to be carried out simultaneously.

In numerical simulations of material failure and structural damage, both microscale and macroscale calculation methods are quite mature. The microscopic scale is typically dominated by atomic interactions and is simulated using molecular dynamics (MD) [17] and Monte Carlo methods [18]. The calculation methods for the macroscopic scale include the finite element method (FEM) [19,20,21] the extended finite element method (XFEM) [22,23,24,25], the edge-based smooth finite element method (ES-FEM) [26,27], etc.

But the connection between the microscopic atomic model and the macroscopic continuum model is the most critical factor affecting the accuracy of the multi-scale computational method. The study of multi-scale coupling computational methods is a frontier and hotspot in the field of materials science. The difficulty lies in two aspects: one is how to achieve the transfer and transition of various physical quantities at different scales and the other is how to achieve the coupling of boundary conditions and computational results at different scales.

As a result, many scholars have proposed different methods. Currently, the main multi-scale analysis methods from micro to macro include: the finite element method combined with atomistic modeling (FEAt) [28], the quasicontinuum method (QC) [29,30,31], the MD-FEM hybrid [32,33,34], the molecular dynamics–cohesive finite element method (MD-CFEM) hybrid [35], the peridynamics method (PD) [36,37,38], the MD-PD method [39], etc.

Among them, the MD-FEM multi-scale method is a hybrid approach that adopts molecular dynamics (MD) in the atomic region and the finite element method (FEM) in the continuum region, respectively. For regions with small deformation and far away from defects, the finite element method is employed based on continuum theory to obtain the corresponding stress and displacement fields. In regions subjected to large deformation or in the vicinity of the concerned defects, the molecular dynamics model is adopted. This approach enables the efficient calculation of macroscopic mechanical parameters such as stress-intensity factor and energy-release rate, while capturing the microscopic deformation mechanisms associated with defect evolution and propagation [15].

This paper mainly uses the MD-FEM multi-scale method to establish a fatigue multi-scale model of AH36 steel from macro to micro, using finite elements and molecular dynamics, to study and analyze the fatigue crack growth process in the heat-affected zone of AH36 steel welding.

## 2. Materials and Methods

### 2.1. Molecular Dynamics Model of AH36 Base Material and Heat-Affected Zone

AH36 steel is a low-carbon steel possessing a polycrystalline microstructure primarily composed of ferrite and pearlite [40]. Its grain size is approximately 1 μm or above, with ferrite serving as the dominant constituent phase. Since the size of the microscopic model to be established is on the order of 15 nm, and ferrite possesses a body-centered cubic (BCC) crystal structure, the microscopic model is therefore simplified to BCC-structured iron crystals in this study. However, single-crystal models exhibit significant anisotropy. To avoid adverse effects of this feature on the accuracy of material parameters derived from simulations, a microscopic model incorporating grain boundaries was established in this work. Specifically, the BCC-Fe single-crystal model was arranged with random crystallographic orientations using the Atomsk tool (https://atomsk.univ-lille.fr/, accessed on 12 October 2023) [41] and packed into a simulation box of predefined dimensions, thus forming a microstructural model containing multiple grain boundaries. The established model is a rectangular prism with dimensions of 150 Å in the x-direction, 150 Å in the y-direction, and 50 Å in the z-direction, containing approximately 96,500 atoms. The x-direction of the model is [100], the y-direction is [010], and the z-direction is [001]. For the visualization analysis of the simulation results, this study adopted the open visualization tool OVITO Basic 3.9.2 [42].

**Figure 2:**
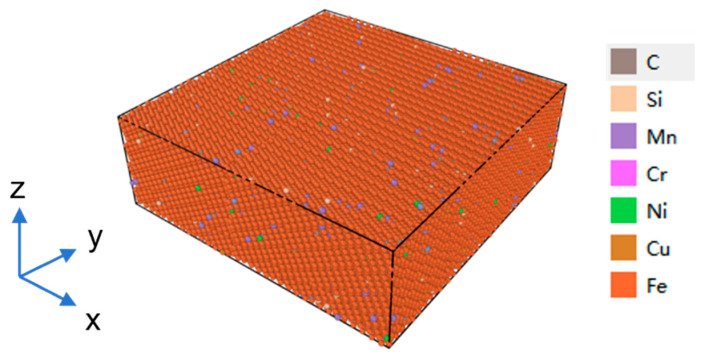
Molecular dynamics model of AH36.

According to the mass fraction of each chemical composition of AH36 steel in ASTM A131/A131M-19 [43] (see Table 1), calculate the atomic ratio of the corresponding element. According to this ratio, some iron atoms in the polycrystalline iron model were randomly replaced with trace elements such as C, Si, Mn, Cr, Ni, Cu, etc. to establish the initial molecular dynamics model of AH36 steel, as shown in Figure 2 (red in the figure represents Fe atoms, and other colors represent alloying elements). In actual materials, alloying elements generally exhibit an inhomogeneous distribution. In this work, this case is simplified to a random and uniform distribution of all elements. Subsequent relaxation simulations were carried out on the model to eliminate spurious internal stresses generated during model construction, driving the atomic system to a thermodynamically equilibrium state with minimum energy and stable temperature. Meanwhile, the lattice structure and interatomic interactions were optimized, thereby guaranteeing the authenticity and reliability of the subsequent simulation results for the mechanical behaviors.

Commonly used interatomic potentials in molecular dynamics simulations include the embedded-atom method (EAM) potential, the modified embedded-atom method (MEAM) potential, and the Lennard–Jones (LJ) potential.

This article will use multi-body potential functions in the forms of MEAM and LJ to describe the interactions between atoms of different elements, as well as monomer potential functions in the forms of EAM and MEAM to describe the interactions between atoms of the same element.

**Figure 3:**
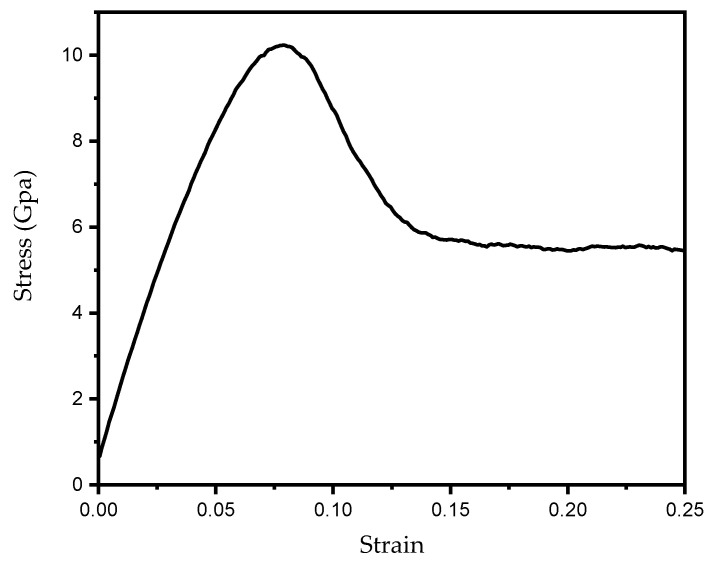
Tensile stress–strain curve of AH36 steel along the [010] y-direction.

The structural model was subjected to a 100 ps relaxation simulation at 300 K using the molecular dynamics software LAMMPS (version 21Nov2023) [44] under the NVT ensemble, thereby ensuring that the system reached a stable configuration. The aforementioned NVT ensemble (canonical ensemble) is a typical equilibrium ensemble in molecular dynamics simulations. For the NVT ensemble, the number of atoms N, system volume V, and temperature T remain constant during the simulation. After relaxation, tensile simulation was performed on the model of AH36 along the y-direction to obtain its stress–strain curve (Figure 3), with a Poisson’s ratio of 0.27 and an elastic modulus of 176.82 GPa.

The elastic modulus of the base metal of AH36 marine steel was experimentally measured as 197.87 GPa. The relative error compared with the simulation result is 10.63%, around 10%, which lies within the allowable error range. Therefore, the elastic modulus obtained from molecular dynamics simulations can be used for multi-scale simulations.

Owing to the size effect in microscopic molecular dynamics simulations, the yield strength reached 10 GPa, which differs considerably from that of the actual material. Accordingly, yield strength was not adopted as a coupling parameter for the multi-scale model.

**Figure 4:**
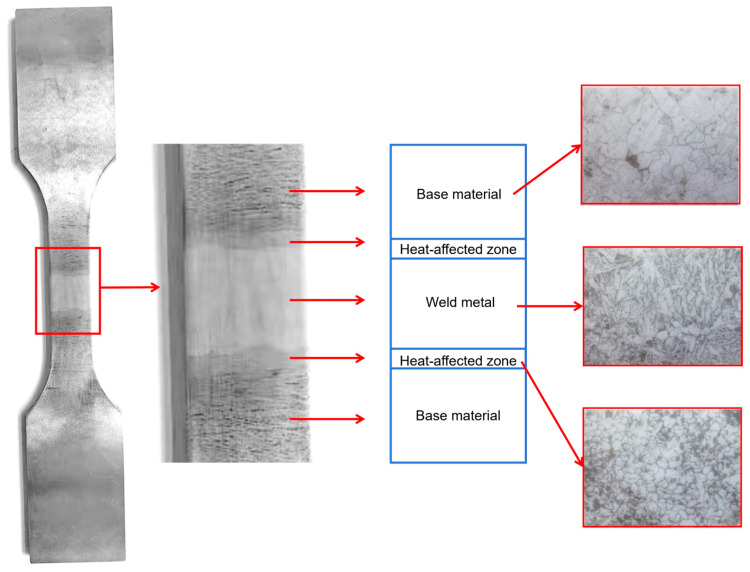
Schematic diagrams of welding fatigue test specimens and different zones, and metallographic micrographs of different zones for AH36 steel.

During the welding process, the microstructure of the material in the region surrounding the weld undergoes alterations due to the temperature elevation, leading to the formation of the heat-affected zone (HAZ). As illustrated in Figure 4, the base metal zone features a coarse crystalline microstructure, the weld zone exhibits a distinctively directional columnar crystalline microstructure, and the heat-affected zone presents a recrystallized microstructure with grain refinement induced by the welding thermal cycle. The microstructures of the three zones differ significantly from each other. As the transition region connecting the base metal and the weld, the heat-affected zone is susceptible to stress concentration under actual loading conditions, which consequently gives rise to damage and failure.

To investigate the damage and failure process of the welded heat-affected zone (HAZ), thermal cycle simulations were conducted on the initial atomic model of AH36 steel under the NVT ensemble in this study, based on the welding thermal cycle characteristics of the region surrounding the weld [40]. The model was heated from 300 K to 950 K and then cooled back to 300 K, thereby establishing a molecular dynamics model for the heat-affected zone of welded AH36 steel.

The thermal cycle process was implemented using the LAMMPS molecular dynamics software, with the detailed simulation procedure as follows: the AH36 steel molecular dynamics model was first steadily relaxed for 10,000 time steps at 300 K. The system temperature was then raised from 300 K to 950 K over 10,000 time steps and held isothermally at 950 K for another 10,000 time steps to enable sufficient structural rearrangement and interatomic interaction of atoms under high temperature. Finally, the model was cooled from 950 K to 300 K within 20,000 time steps, followed by further steady relaxation for 20,000 time steps at 300 K. The time step was set to 0.001 ps throughout the entire thermal cycle. The peak temperature was maintained below the melting point of Fe during the heating and cooling process, which reproduces the formation of the HAZ caused by the thermal cycle at the weld of AH36 steel and its influence on the surrounding microstructure. Accordingly, the goal of constructing a molecular dynamics model for the HAZ of AH36 steel was fulfilled.

**Figure 5:**
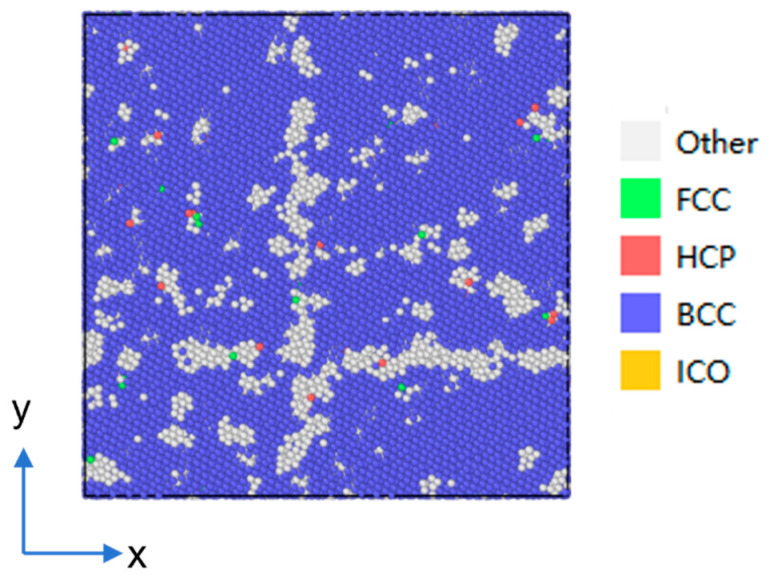
Schematic diagram of the molecular dynamics model of the heat-affected zone of AH36 steel (where blue atoms are BCC structures and white atoms are disordered structures).

Following the above heating–cooling simulation, the molecular dynamics model of the heat-affected zone (HAZ) of AH36 high-strength steel is obtained, as illustrated in Figure 5. This figure shows the schematic microstructure characterized by common neighbor analysis (CNA), where different colors denote distinct crystal structures. The “Other” atoms marked in white represent the atomic group that cannot be identified as ideal local crystal structures of BCC, FCC, HCP, or icosahedral (ICO) by common neighbor analysis (CNA). Such atoms are mainly distributed at grain boundaries, dislocation cores, free surfaces, and locally disordered regions. Within the BCC matrix of AH36 steel, the distribution and morphology of these atoms directly reflect the defect density and disorder level of the HAZ microstructure formed after the welding thermal cycle, providing key atomic-scale information for characterizing thermally induced micro-damage.

**Figure 6:**
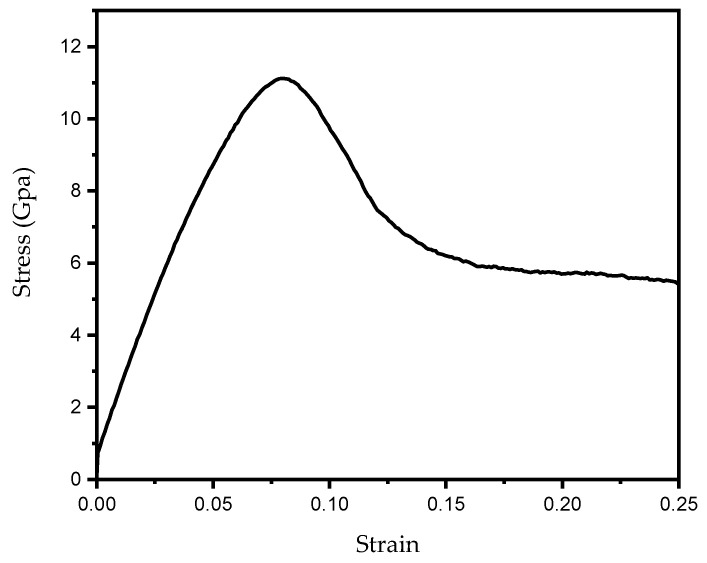
Tensile stress–strain curve of the heat-affected zone of AH36 steel along the [010] y-direction.

Tensile simulations along the y-direction were carried out on the heat-affected zone (HAZ) model using LAMMPS, and the corresponding stress–strain curve was acquired (see Figure 6). The elastic modulus is 182.07 GPa and the Poisson’s ratio of 0.27 is consistent with that of the base metal.

The elastic modulus of the HAZ of AH36 marine steel was experimentally measured as 190.53 GPa, and the relative error compared with the simulation results is 7.20%, which is less than 10% and falls within the allowable error range. Therefore, the elastic modulus of the HAZ obtained from molecular dynamics simulations can be employed for multi-scale simulations.

The yield strength of the HAZ also presents the size effect at the nanoscale, exceeding 10 GPa, which shows a considerable deviation from that of the actual material. Accordingly, this yield strength is not adopted as a coupling parameter for the multi-scale model either.

### 2.2. Multi-Scale Model of AH36 Under Fatigue Load

**Figure 7:**
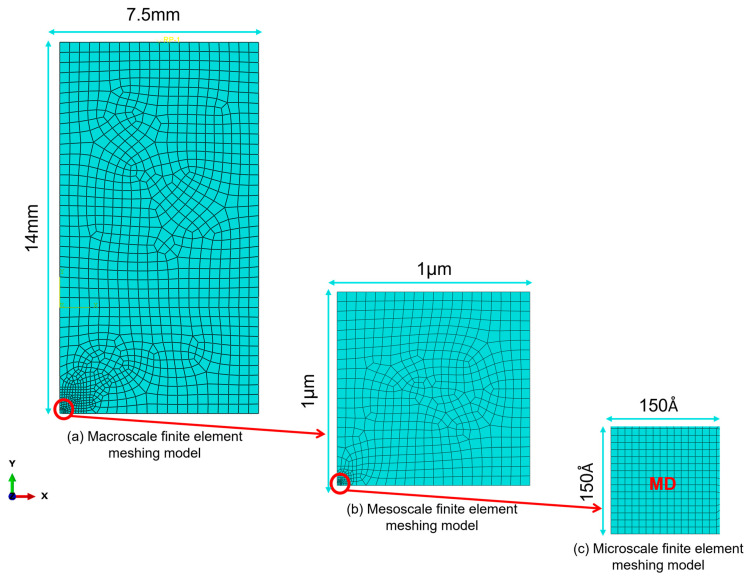
Schematic diagram of the dimensions and meshing of macroscopic, mesoscopic and microscopic regions.

The fatigue crack propagation process in materials involves three distinct stages, namely, initiation, propagation, and instability, which correspond to three spatial scales—microscale, mesoscale, and macroscale. Accordingly, the cross-scale fatigue model is divided into three submodels corresponding to these scales, as illustrated in Figure 7. Specifically, the macroscopic model corresponds to the millimeter scale (greater than 1 mm), the mesoscopic model to the micrometer scale (ranging from 100 nm to 1 mm), and the microscopic model to the nanometer scale (less than 100 nm) [14].

The main function of the macroscopic and mesoscopic models is to provide boundary conditions, namely, boundary displacements under fatigue loads, for the microscopic simulation of fatigue cracks based on molecular dynamics. Among them, the macroscopic and mesoscopic models are simulated by the finite element method, and the fatigue tension–pull cycle loading is simplified to half a cycle to extract the peak displacement state. After the displacement condition is passed to the microscopic model, a complete fatigue-cycle simulation is carried out based on molecular dynamics methods.

After the microscopic molecular dynamics model completes the fatigue simulation, the obtained information such as crack morphology is transmitted and updated to the mesoscopic finite element model. After updating the crack morphology, the mesoscopic model recalculates through simulation to obtain a new set of boundary conditions, which are then transferred back to the microscopic model. The microscopic model then performs the fatigue-cycle simulation again. The above simulation procedure is repeated iteratively between the microscopic and mesoscopic models, thus accomplishing the multi-scale cyclic-coupling simulation of fatigue crack propagation.

#### 2.2.1. Establishment of the Macroscopic Model and Its Multi-Scale Coupling Method

The macroscopic model was established and analyzed via the Abaqus (2020) finite element software. According to the dimensions of conventional welded specimens adopted in fatigue tests (see Figure 4), a macroscopic model consisting of the base metal and the heat-affected zone (HAZ) was constructed. Given that this study mainly focuses on fatigue crack propagation in the HAZ, a half-model containing the base metal and the HAZ on one side of the specimen was used for simplification during modeling.

**Figure 8:**
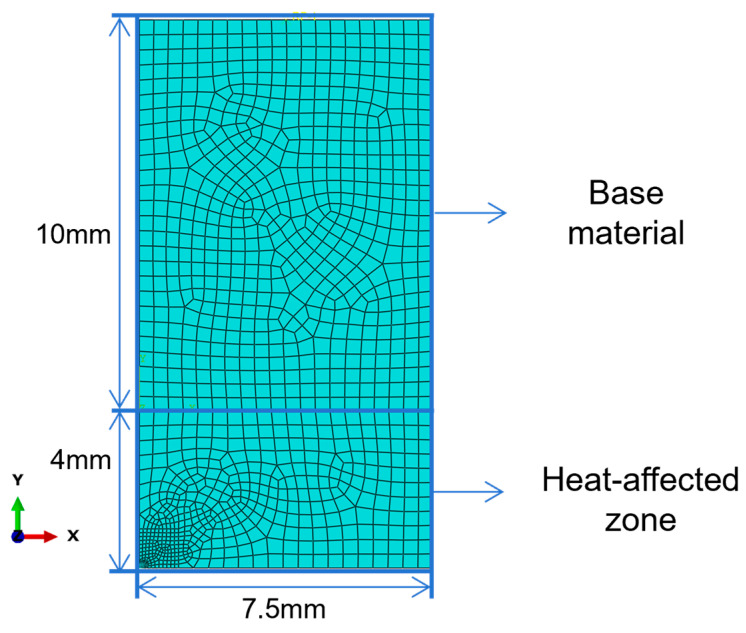
Macroscopic finite element model plane dimensions and regional diagram.

In the macroscopic model (shown in Figure 8), the base metal zone is assigned dimensions of 7.5 mm in the x-direction, 10 mm in the y-direction, and 7.6 mm in the z-direction. The heat-affected zone (HAZ) is set to 7.5 mm in the x-direction, 4 mm in the y-direction, and 7.6 mm in the z-direction.

The material parameters used for both the base metal zone and the HAZ are the elastic modulus and Poisson’s ratio derived from molecular dynamics simulations. A tie constraint is applied between the two regions, resulting in no relative displacement across the contact interface and a fully rigid bonding. The mesh type adopted is hexahedral swept mesh.

The lower boundary of the model is fixed, and a peak load of 350 MPa is applied to the upper boundary, corresponding to half-cycle loading in a tension–tension fatigue cycle. The strain field and displacement field of the model at the peak load state are thus obtained through simulation. The peak load is lower than the experimentally measured yield strength of AH36 marine steel, ensuring that the simulation of the macroscopic model is carried out within the elastic range.

The multi-scale coupling from the macroscopic model to the mesoscopic model is mainly realized by transferring boundary displacements. To achieve such displacement transfer, hierarchical local mesh refinement is conducted in the macroscopic model. A region with a mesh size identical to the dimension of the mesoscopic model is eventually obtained, and the nodal displacements of all elements within this region are averaged. The averaged result is then adopted as the boundary condition for the mesoscopic model, thus completing the multi-scale coupling.

**Figure 9:**
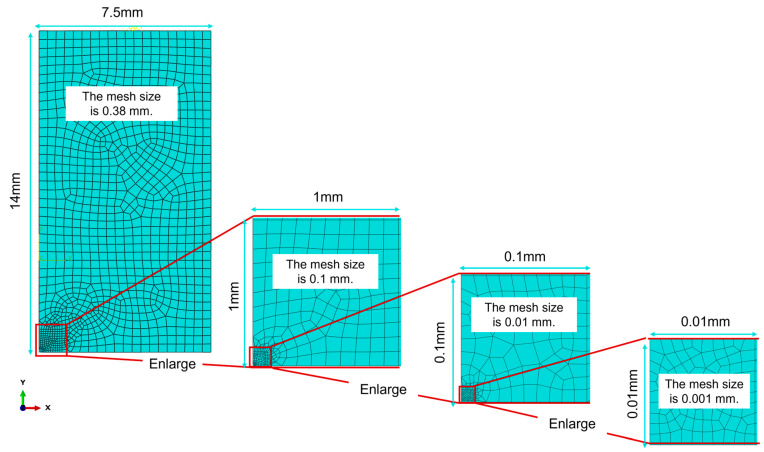
Schematic diagram of mesh refinement for the macroscopic model.

Since the multi-scale simulation is constructed for the heat-affected zone (HAZ), hierarchical local mesh refinement is applied to the HAZ. The detailed mesh refinement procedure is shown in Figure 9. The mesh is finally refined to 0.001 mm (i.e., 1 μm), consistent with the size of the mesoscopic model. The nodal displacements inside the region with a mesh size of 0.001 mm are averaged, and the calculated result is 0.051095 μm.

To ensure the accuracy of the simulation results, the mesh in the region with a size of 0.001 mm was further refined to 0.0005 mm. The above simulation and calculation procedure was repeated, yielding an average nodal displacement of 0.051979 μm. The error relative to the result before mesh refinement is only 1.73%. Therefore, the average displacement calculated from the nodes in the region with a mesh size of 0.001 mm can be adopted as the boundary condition for the mesoscopic model.

Given the considerable scale difference between the macroscopic and microscopic models, the boundary conditions transmitted from the macroscopic model to the mesoscopic model are barely affected by the feedback of the microscopic model. Thus, the boundary conditions provided by the macroscopic model to the mesoscopic model are kept constant in the subsequent cyclic multi-scale coupling between the mesoscopic and microscopic models.

#### 2.2.2. Establishment of the Mesoscopic Model and Its Multi-Scale Coupling Method

The mesoscopic model, acting as the intermediate scale bridging macroscopic and microscopic simulations, is established using the ABAQUS finite element software. Since the mesoscopic model lies within HAZ, its material parameters are assigned as the elastic modulus and Poisson’s ratio of the HAZ derived from molecular dynamics simulations. The geometric dimensions of the mesoscopic model (see Figure 7b) are 1 μm in the x-direction, 1 μm in the y-direction, and 0.005 μm in the z-direction, where the z-direction length is kept consistent with that of the microscopic model. The mesh type used is hexahedral swept mesh. The lower boundary of the mesoscopic model is fixed, and a displacement condition transferred from the macroscopic model through multi-scale coupling is applied to the upper boundary, corresponding to the half-cycle peak displacement in tension–tension fatigue cycles with a magnitude of 0.05 μm.

The multi-scale coupling from the mesoscopic model to the microscopic model is mainly realized by transferring boundary conditions. To achieve the transfer of such boundary conditions, a region with the same size as the microscopic model is defined in the mesoscopic model. This region is treated as the finite element microscopic model and is further refined by hierarchical mesh refinement. The average displacement of the boundary nodes in this region is computed, and the strain value of the region is derived according to the strain calculation Equation (1):

(1) ε=∆l/l

In the above equation, ε denotes the strain, ∆l represents the variation in length, and l stands for the original length. This strain value is then applied as the boundary condition for the molecular dynamics microscopic model, by which the multi-scale coupling is accomplished.

**Figure 10:**
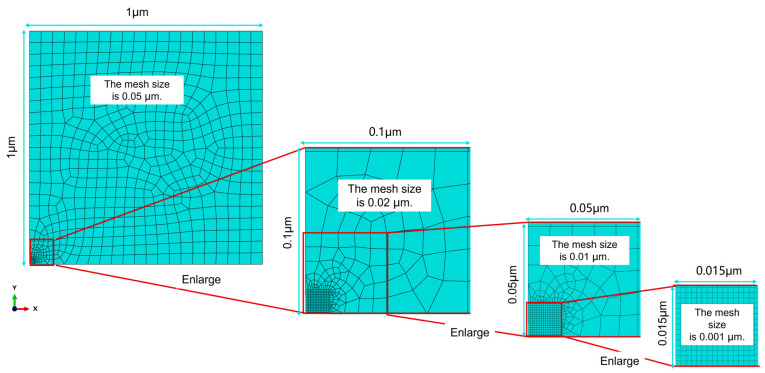
Schematic diagram of mesh refinement for the microscopic model.

The detailed hierarchical mesh refinement process is illustrated in Figure 10, and the mesh is eventually refined to 0.001 μm. The region with a mesh size of 0.001 μm is exactly the microscopic model region defined on the mesoscopic model. After averaging the boundary node displacements and computing the strain for this region, the strain value is determined to be 0.077755.

To ensure the accuracy of the simulation results, the mesh size in the microscopic model region was further refined to 0.0005 μm, and the above simulation and calculation were repeated. The strain of this region was determined to be 0.077948, with a relative error of only 0.25% compared with the result before mesh refinement. Therefore, the calculated result corresponding to a mesh size of 0.001 μm can be transferred to the microscopic model as the boundary condition for molecular dynamics simulations.

#### 2.2.3. Establishment of the Microscopic Model and Its Multi-Scale Coupling Method

**Figure 11:**
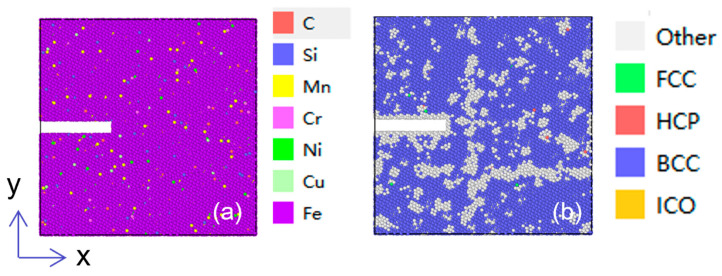
Molecular dynamics microscopic model: (**a**) elemental distribution diagram; (**b**) crystal structure diagram.

The initiation and propagation of fatigue cracks mostly originate from initial defects, such as various micro-defects including vacancies, voids, inclusions, and microcracks [45]. Accordingly, a square initial defect is introduced at the midpoint of the left boundary of the established microscopic molecular dynamics model for the HAZ of AH36 steel, with dimensions of 50 Å in the *x*-direction, 10 Å in the *y*-direction, and 50 Å in the *z*-direction, as illustrated in Figure 11. The selection of the initial defect exerts a certain influence on the crack initiation stage but has no significant effect on the investigation of laws such as crack propagation mechanism, morphology evolution, and void behavior.

Fatigue loading is implemented using the deform command in LAMMPS, with a strain rate of 1 × 10^10^ s^−1^ and a strain ratio maintained at approximately 0.1, to perform tension–tension fatigue simulations under a positive load ratio. A negative load ratio corresponds to tension–compression fatigue cyclic loading. The occurrence of compressive stress increases the crack propagation rate and affects the evolution of crack morphology as well as the nucleation and growth of voids. Since this study mainly focuses on multi-scale crack propagation simulations under tension–tension fatigue cyclic loading, the stress ratio is set to a positive value. The peak strain adopts the strain value transferred from the mesoscopic model. All subsequent microscopic model simulations are conducted at a temperature of 300 K, with the NVT ensemble and periodic boundary conditions applied.

The multi-scale coupling from the microscopic model to the mesoscopic model mainly consists of two components: the equivalence treatment of crack morphology and crack tip tracking.

1. Crack morphology equivalence

After the microscopic model has completed fatigue simulation for a certain number of cycles, crack propagation and morphology evolution take place. At this stage, the updated crack morphology needs to be equivalently fed back to the mesoscopic model. The mesoscopic model is then recalculated to obtain the new boundary strain conditions for the microscopic model, and the molecular dynamics fatigue simulation of the microscopic model is continued with the new boundary strain conditions, thus accomplishing the cyclic multi-scale coupling between the microscopic and mesoscopic models.

**Figure 12:**
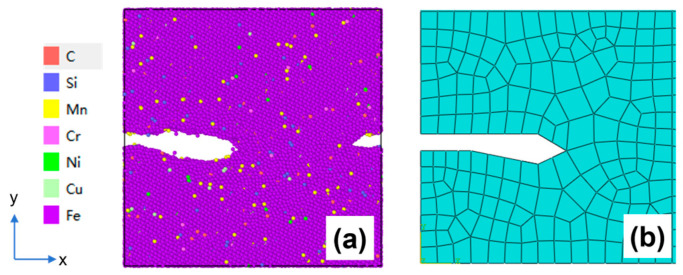
(**a**) Crack propagation diagram of the microscopic molecular dynamics model; (**b**) microscopic region map of the crack equivalent to a mesoscopic finite element model.

The specific procedure for the equivalence treatment of crack morphology is to simplify the simulated crack morphology from the molecular dynamics microscopic model into a basic geometric polygon and map it equivalently to the microscopic model region defined in the mesoscopic model by means of coordinate point mapping so as to update the mesoscopic model (Figure 12).

Due to the influence of periodic boundary conditions in molecular dynamics simulations, when the left crack in Figure 12a propagates along the *x*-direction, part of its structure passes through the left boundary and re-enters the simulation box from the right boundary, which visually appears as a crack on the right side. This phenomenon arises from the boundary effect in numerical simulations rather than representing actual double-sided cracks in the material. Therefore, the complete crack morphology obtained from the simulation is composed of the left crack together with the right crack. Accordingly, the complete crack morphology formed by combining the left and right cracks should be transferred when equivalently transmitting the crack morphology to the mesoscopic model.

2. Crack tip tracking

**Figure 13:**
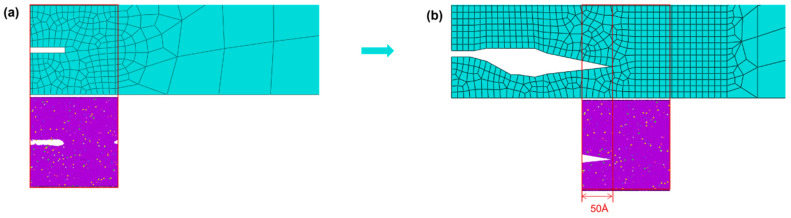
Schematic diagram of crack tip transformation zone: (**a**) initial crack tip configuration with the corresponding microscopic model; (**b**) redefined crack tip region (including 50 Å ahead of the crack tip) and reconstructed microscopic model.

As the crack propagates forward continuously, the microscopic model needs to be always centered on the crack tip region. Therefore, it is necessary to redefine the microscopic model region on the mesoscopic model and reconstruct the molecular dynamics microscopic model according to the simplified shape of the crack tip, as illustrated in Figure 13. The redefined microscopic model region should include the segment 50 Å ahead of the crack tip, as shown in Figure 13b. Meanwhile, when transferring boundary conditions to the new molecular dynamics microscopic model, the boundary nodal displacements of the redefined microscopic model region in the mesoscopic model should be extracted correspondingly. The remaining part of the crack is preserved in the mesoscopic model as a boundary with its geometry unchanged.

## 3. Multi-Scale Crack Propagation Simulation Results

In this study, a total of 600 fatigue cycles were simulated. After every 50 tension–tension fatigue loading cycles completed by the microscopic model, one cyclic multi-scale coupling with the mesoscopic model was performed.

During this process, 12 information exchanges were carried out between the microscopic and mesoscopic models, and 9 replacements of the microscopic region were implemented to track the crack tip, as illustrated in Figure 14, Figure 15, Figure 16, Figure 17, Figure 18, Figure 19, Figure 20, Figure 21, Figure 22, Figure 23, Figure 24 and Figure 25.

**Figure 14:**
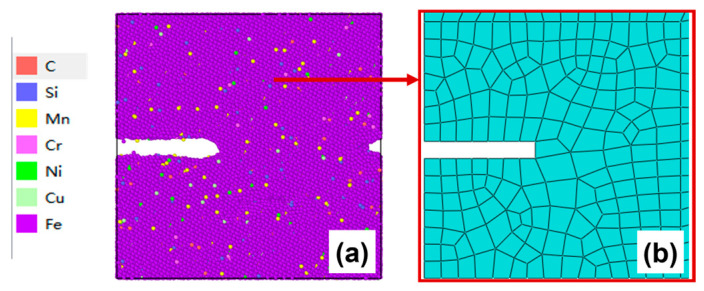
(**a**) Molecular dynamics model after 0–50 cycles; (**b**) equivalent crack morphology after 0–50 cycles (local).

The crack morphology after 0–50 loading cycles is shown in Figure 14a. The result of simplifying and mapping this crack morphology back to the microscopic model region defined in the mesoscopic model is presented in Figure 14b. In this interaction, only the crack morphology was updated, and no replacement of the microscopic region was conducted.

**Figure 15:**
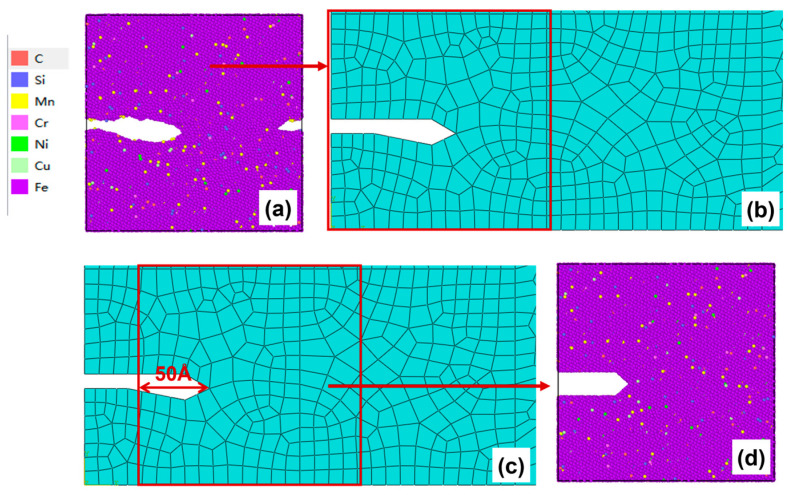
(**a**) Microcrack morphology after 50–100 cycles; (**b**) crack morphology equivalent to the mesoscopic model after 50–100 cycles; (**c**) schematic diagram of microregion transition after 50–100 cycles (50 Å before the crack tip); (**d**) a microscopic model based on the new area after 50–100 cycles.

After 50–100 cycles, the crack morphology of the microscopic model is shown in Figure 15a. The modified crack morphology was simplified and mapped back to the microscopic model region defined in the mesoscopic model, and the result is presented in Figure 15b. Subsequently, the first replacement of the microscopic region was carried out: a region 50 Å ahead of the crack tip was chosen as the new microscopic model region (Figure 15c). A new molecular dynamics microscopic model was then constructed accordingly (Figure 15d) for the subsequent cyclic loading.

**Figure 16:**
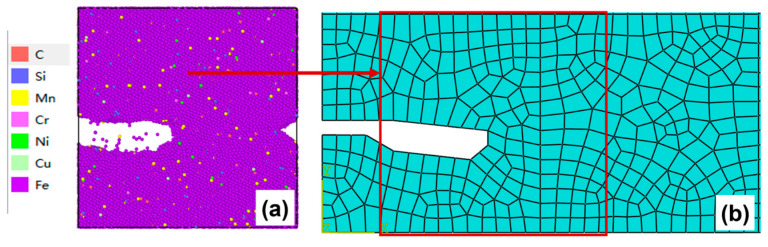
(**a**) Molecular dynamics model at the end of 100–150 cycles; (**b**) equivalent crack morphology (local) after 100–150 cycles.

After 100–150 cycles, the crack morphology of the microscopic model is shown in Figure 16a. After the modified crack morphology was simplified and mapped back to the mesoscopic model, the updated crack morphology is presented in Figure 16b. No replacement of the microscopic region was conducted in this interaction.

**Figure 17:**
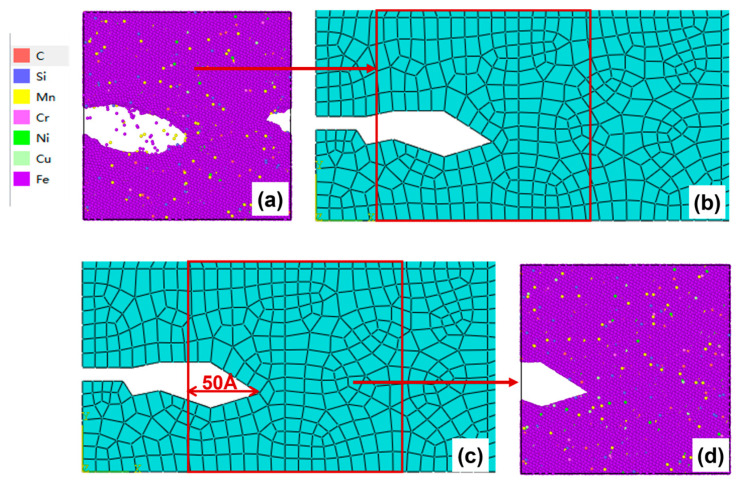
(**a**) Microcrack morphology after 150–200 cycles; (**b**) crack morphology equivalent to the mesoscopic model after 150–200 cycles; (**c**) schematic diagram of microregion transition after 150–200 cycles (50 Å before the crack tip); (**d**) a microscopic model based on the new area after 150–200 cycles.

After 150–200 cycles, the crack morphology of the microscopic model is shown in Figure 17a. After the modified crack morphology was simplified and mapped back to the mesoscopic model, the updated crack morphology is presented in Figure 17b. Subsequently, the second replacement of the microscopic region was performed (Figure 17c), and a new molecular dynamics microscopic model was established accordingly (Figure 17d) for the subsequent cyclic loading.

**Figure 18:**
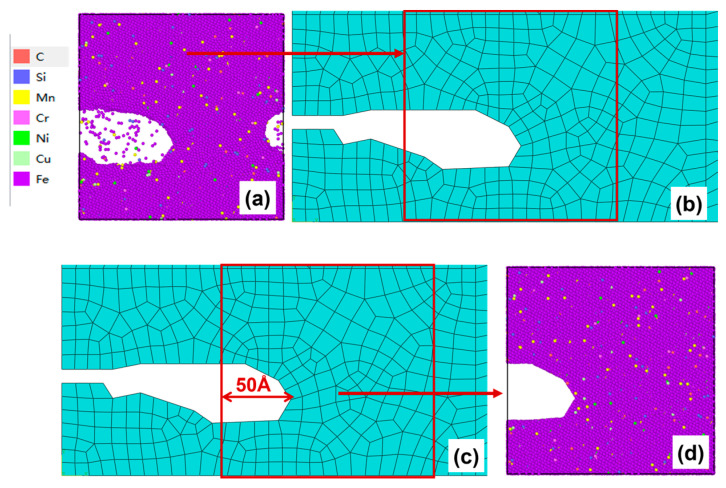
(**a**) Microcrack morphology after 200–250 cycles; (**b**) Crack morphology equivalent to the mesoscopic model after 200–250 cycles; (**c**) Schematic diagram of microregion transition after 200–250 cycles (50 Å before the crack tip); (**d**) A microscopic model based on the new area after 200–250 cycles.

After 200–250 cycles, the crack morphology of the microscopic model is shown in Figure 18a. After the modified crack morphology was simplified and mapped back to the mesoscopic model, the updated crack morphology is presented in Figure 18b. Subsequently, the third replacement of the microscopic region was performed (Figure 18c), and a new molecular dynamics microscopic model was established accordingly (Figure 18d) for the subsequent cyclic loading.

**Figure 19:**
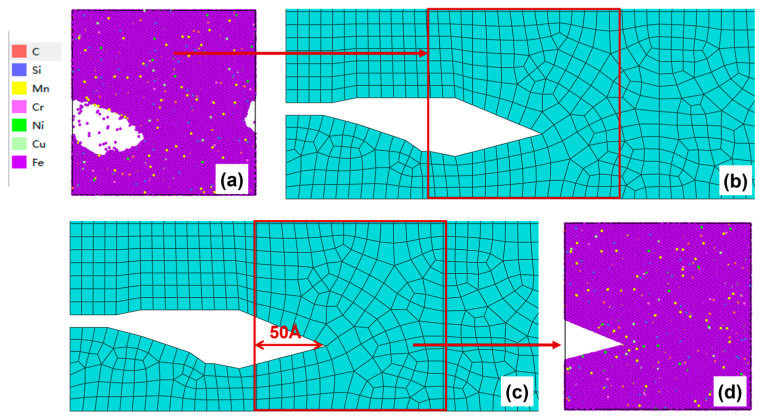
(**a**) Microcrack morphology after 250–300 cycles; (**b**) crack morphology equivalent to the mesoscopic model after 250–300 cycles; (**c**) Schematic diagram of microregion transition after 250–300 cycles (50 Å before the crack tip); (**d**) a microscopic model based on the new area after 250–300 cycles.

After 250–300 cycles, the crack morphology of the microscopic model is shown in Figure 19a. After the modified crack morphology was simplified and mapped back to the mesoscopic model, the updated crack morphology is presented in Figure 19b. Subsequently, the fourth replacement of the microscopic region was performed (Figure 19c), and a new molecular dynamics microscopic model was established accordingly (Figure 19d) for the subsequent cyclic loading

**Figure 20:**
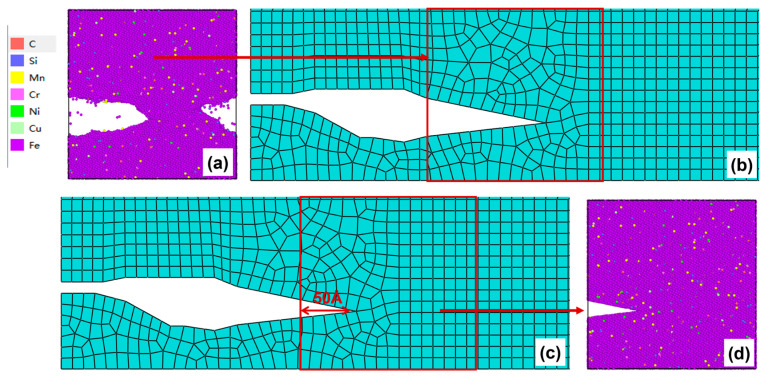
(**a**) Microcrack morphology after 300–350 cycles; (**b**) crack morphology equivalent to the mesoscopic model after 300–350 cycles; (**c**) schematic diagram of microregion transition after 300–350 cycles (50 Å before the crack tip); (**d**) a microscopic model based on the new area after 300–350 cycles.

After 300–350 cycles, the crack morphology of the microscopic model is shown in Figure 20a. After the modified crack morphology was simplified and mapped back to the mesoscopic model, the updated crack morphology is presented in Figure 20b. Subsequently, the fifth replacement of the microscopic region was performed (Figure 20c), and a new molecular dynamics microscopic model was established accordingly (Figure 20d) for the subsequent cyclic loading.

**Figure 21:**
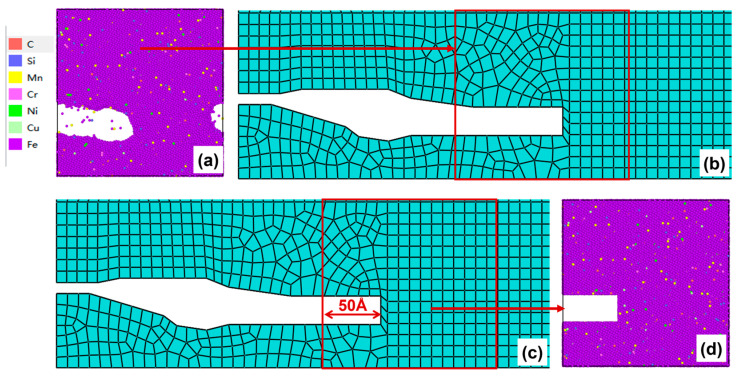
(**a**) Microcrack morphology after 350–400 cycles; (**b**) crack morphology equivalent to the mesoscopic model after 350–400 cycles; (**c**) schematic diagram of microregion transition after 350–400 cycles (50 Å before the crack tip); (**d**) a microscopic model based on the new area after 300–350 cycles.

After 350–400 cycles, the crack morphology of the microscopic model is shown in Figure 21a. After the modified crack morphology was simplified and mapped back to the mesoscopic model, the updated crack morphology is presented in Figure 21b. Subsequently, the sixth replacement of the microscopic region was performed (Figure 21c), and a new molecular dynamics microscopic model was established accordingly (Figure 21d) for the subsequent cyclic loading.

**Figure 22:**
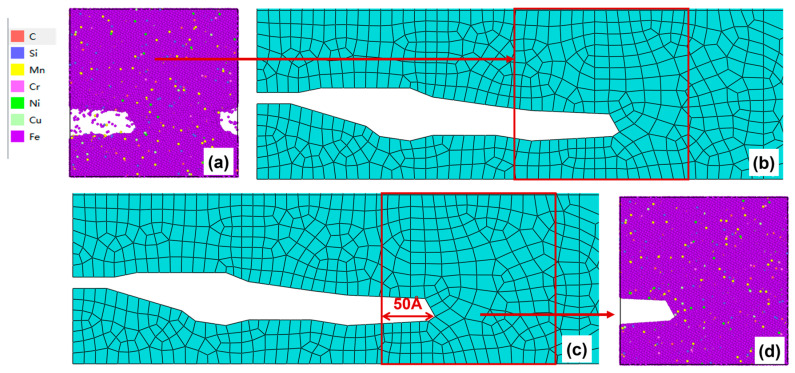
(**a**) Microcrack morphology after 400–450 cycles; (**b**) crack morphology equivalent to the mesoscopic model after 400–450 cycles; (**c**) schematic diagram of microregion transition after 400–450 cycles (50 Å before the crack tip); (**d**) a microscopic model based on the new area after 400–450 cycles.

After 400–450 cycles, the crack morphology of the microscopic model is shown in Figure 22a. After the modified crack morphology was simplified and mapped back to the mesoscopic model, the updated crack morphology is presented in Figure 22b. Subsequently, the seventh replacement of the microscopic region was performed (Figure 22c), and a new molecular dynamics microscopic model was established accordingly (Figure 22d) for the subsequent cyclic loading.

**Figure 23:**
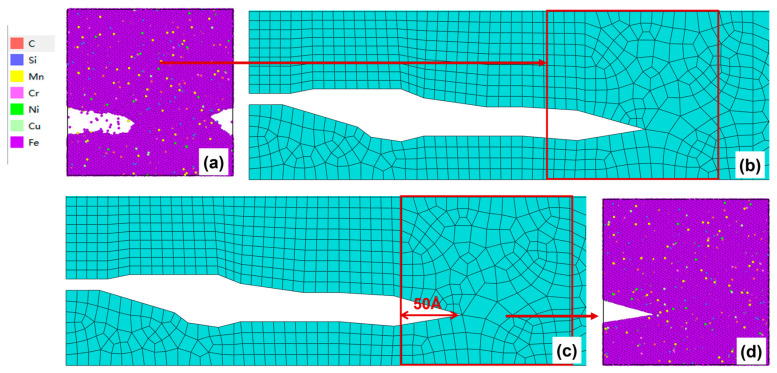
(**a**) Microcrack morphology after 450–500 cycles; (**b**) crack morphology equivalent to the mesoscopic model after 450–500 cycles; (**c**) schematic diagram of microregion transition after 450–500 cycles (50 Å before the crack tip); (**d**) a microscopic model based on the new area after 450–500 cycles.

After 450–500 cycles, the crack morphology of the microscopic model is shown in Figure 23a. After the modified crack morphology was simplified and mapped back to the mesoscopic model, the updated crack morphology is presented in Figure 23b. Subsequently, the eighth replacement of the microscopic region was performed (Figure 23c), and a new molecular dynamics microscopic model was established accordingly (Figure 23d) for the subsequent cyclic loading.

**Figure 24:**
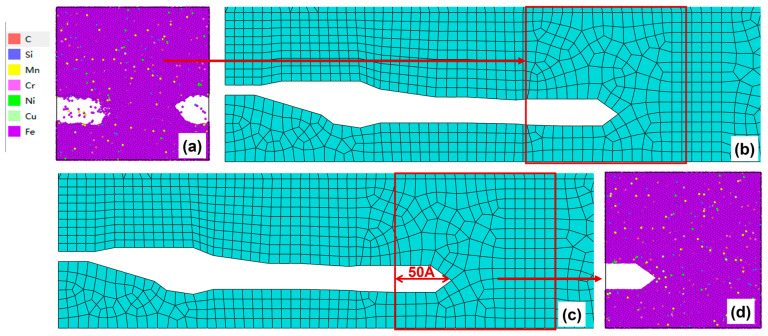
(**a**) Microcrack morphology after 500–550 cycles; (**b**) crack morphology equivalent to the mesoscopic model after 500–550 cycles; (**c**) schematic diagram of microregion transition after 500–550 cycles (50 Å before the crack tip); (**d**) a microscopic model based on the new area after 500–550 cycles.

After 500–550 cycles, the crack morphology of the microscopic model is shown in Figure 24a. After the modified crack morphology was simplified and mapped back to the mesoscopic model, the updated crack morphology is presented in Figure 24b. Subsequently, the ninth replacement of the microscopic region was performed (Figure 24c), and a new molecular dynamics microscopic model was established accordingly (Figure 24d) for the subsequent cyclic loading.

**Figure 25:**
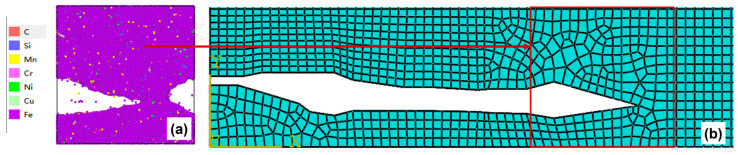
(**a**) Microcrack morphologies after 550–600 cycles; (**b**) crack morphology equivalent to the mesoscopic model after 550–600 cycles.

After 550–600 cycles, the crack morphology of the microscopic model is shown in Figure 25a. After the modified crack morphology was simplified and mapped back to the mesoscopic model, the updated crack morphology is presented in Figure 25b.

During the fatigue loading simulation, atomic scattering occurred inside the crack. The main reason for this is that under the action of cyclic loading, local high stress was generated at the crack tip and its nearby area due to stress concentration. With the accumulation of fatigue damage, the original lattice structure gradually became unstable and was destroyed. The interatomic force changed from strong attraction to weak interaction or even mutual repulsion, and the atomic spacing continuously increased. Thus, at the microscopic level, it was manifested as the discrete distribution of atoms.

After 0 to 600 cycles of loading, the length of the crack along the x-direction expanded from 50 Å to 469 Å. The crack extension length per 50 cycles of loading, the total crack length, the peak boundary strain caused by cyclic loading in the MD region, and the crack propagation speed are shown in Table 2.

## 4. Analysis of Microstructural Evolution During Crack Propagation

### 4.1. Crack Sharpening and Passivation

During fatigue crack propagation, sharpening means the crack tip becomes sharp with an increase in stress concentration, which favors the rapid propagation of cracks. In contrast, blunting indicates that the crack tip is rounded due to plastic deformation or chemical dissolution, leading to a reduction in stress concentration. This suppresses or slows down the crack propagation rate, and may simultaneously cause crack deflection, branching, width enlargement and other morphological changes.

**Figure 26:**
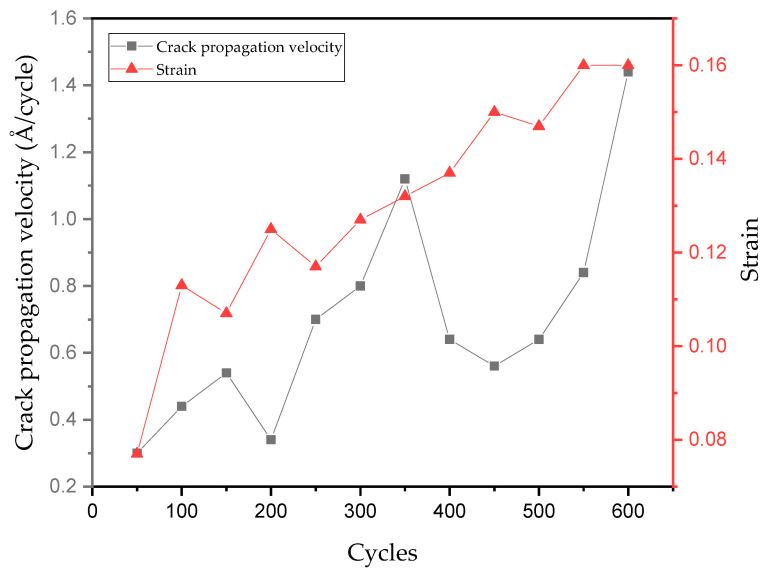
The variation in crack propagation velocity and peak boundary strain with the number of loading cycles.

**Figure 27:**
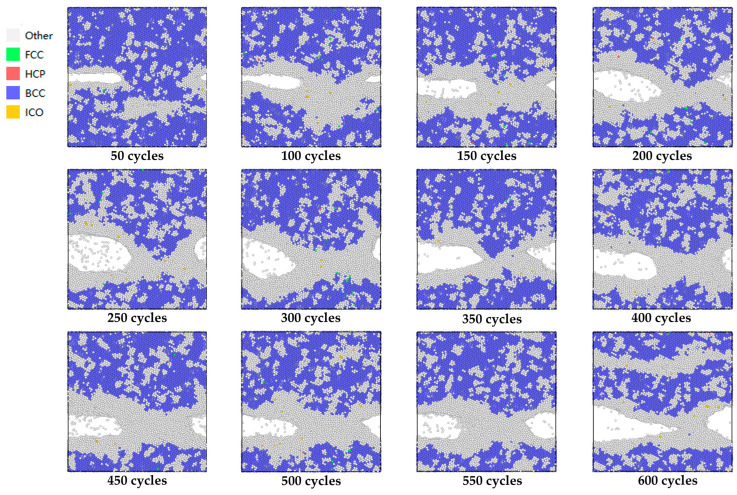
Schematic diagram of crack tip from 0 to 600 cycles.

Under cyclic loading from 0 to 600 cycles, the variation in crack propagation velocity and peak boundary strain with the number of loading cycles is illustrated in Figure 26, while the evolution of crack tip morphology during these 600 cycles is depicted in Figure 27.

Under the action of these 600 cycles of loading, the crack tip obviously underwent passivation, which occurred respectively between 100 and 150 cycles, 200 and 250 cycles, and 350 and 400 cycles. After 150 and 400 cycles, a decrease in crack propagation velocity was observed. From 150 to 200 cycles, the propagation velocity decreased from 27Å per 50 cycles to 17Å, and from 400 to 450 cycles, it decreased from 32Å per 50 cycles to 28Å. This phenomenon indicates that under fatigue-cycle loads, there is indeed passivation at the tip of the microcrack, and passivation reduces the propagation velocity of the microcrack to a certain extent.

Secondly, as shown in Figure 26, the crack propagation velocity decreases between 150 and 200 cycles. Combined with the analysis in Figure 27, it can be found that during the 50 cycles, the crack undergoes longitudinal propagation; that is, the crack morphology widens, hindering the crack’s forward growth and thus resulting in a decrease in the crack propagation velocity.

Under the same peak boundary displacement, the crack propagation velocity increased significantly during the 550–600 cycles compared to the 500–550 cycles, and the crack propagation length increased from 42Å to 73Å. Combined crack morphology comparison analysis (see Figure 27) reveals significant sharpening at the crack tip, which is the main reason for the increase in crack propagation velocity during the 550–600 cycles.

It can be seen from Figure 26 that the peak boundary strain is not the only factor affecting the crack propagation rate. An increase in the peak strain does not necessarily lead to a rise in the crack propagation rate, nor does a decrease in the peak strain necessarily result in a decline in the rate, thus giving rise to the phenomenon of crack propagation retardation. A comprehensive analysis is required by taking into account the crack morphology, the blunting and sharpening of the crack during cyclic loading, and the crack propagation direction. Meanwhile, this phenomenon is consistent with the overload retardation behavior of fatigue crack propagation obtained by Liang Yuebing et al. [13] in fatigue crack experiments on AH36 steel.

### 4.2. Perforations

**Figure 28:**
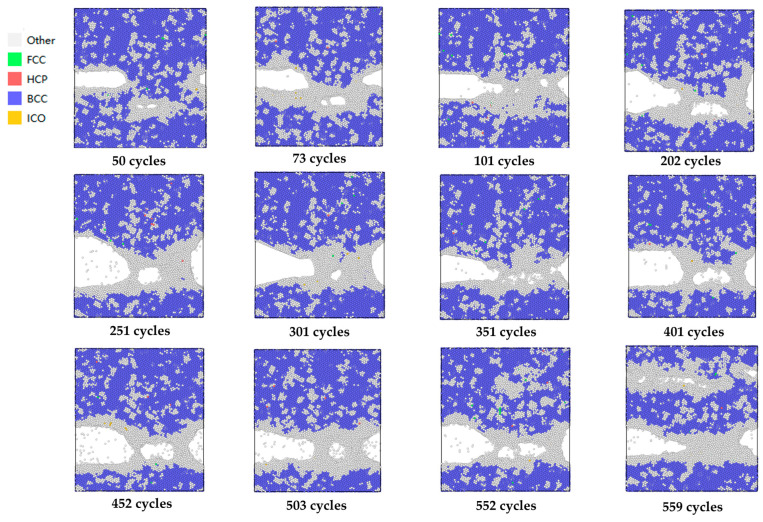
The appearance of voids near the crack tip within 0 to 600 cycles.

During the 600 cycles of the multi-scale simulation of crack propagation, varying numbers of voids appeared at and near the crack tip every 50 cycles, as shown in Figure 28. A single void emerged near the crack tip at the 201st, 251st, 301st, 401st, 452nd and 503rd cycles; two voids appeared near the crack tip at the 50th, 73rd, 101st and 552nd cycles; multiple voids were observed near the crack tip at the 351st cycle. At the 559th cycle, multiple voids also appeared at a position approximately 70 Å away from the crack in the height direction, and these voids aggregated and coalesced along the x-direction. It is found that all voids occurred in regions where other-type atoms accumulated, and the aggregation of other-type atoms also appeared at and near the crack tip. The differences in the size and number of voids correspond to the degree of micro-damage accumulation to a certain extent. With the increase in the number of fatigue cycles and the peak strain, both the number and size of the voids increased, which also reflects the aggravation of micro-damage accumulation.

**Figure 29:**
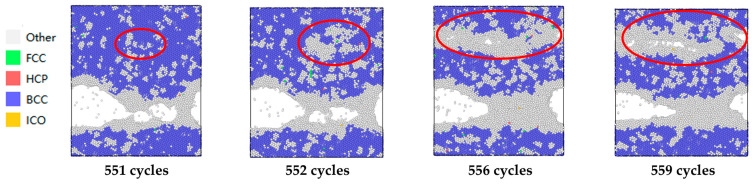
Grain boundary convergence and void formation connectivity in cycles 551 to 559.

During the 551st to 559th cycles, as shown in Figure 29, it can be observed that the aggregation of discrete other-type atom regions occurs first. With further cyclic loading, the other-type atom regions gradually expand, accompanied by the emergence of voids. These voids then begin to aggregate and coalesce to form transverse microcracks.

Therefore, it can be inferred that during fatigue crack propagation, plastic deformation at the crack tip induced by stress concentration first alters the interatomic lattice structure, resulting in an increase in other-type atoms and their aggregation at and near the plastically deformed crack tip. The disorder and instability of the interatomic structure make the regions with other-type atom aggregation more susceptible to void formation, and the nucleation, aggregation and coalescence of voids promote the initiation and propagation of cracks to a certain extent.

However, as shown in Figure 27, with further fatigue cyclic loading and the continuous accumulation of plastic deformation at the crack tip, some voids either aggregate, coalesce and fuse with the main crack or undergo healing. As a result, after the completion of fatigue cyclic loading, the positions where voids originally appeared are transformed into other-type atom regions. The healing of voids reduces the crack propagation rate to a certain extent, which may be one of the reasons why the crack propagation rate remains at a low level in the initial stage of microcrack nucleation.

The nucleation and evolution of voids observed in atomic-scale molecular dynamics simulations arise from the aggregation of atomic vacancies and local plastic deformation under stress concentration. These are natural numerical outcomes driven by physical mechanisms rather than numerical artifacts. Due to the extreme difficulty of in situ dynamic observation of nanoscale voids under fatigue cycling, there is still a lack of experimental evidence directly corresponding to the atomic simulations. Nevertheless, this phenomenon is consistent with the well-accepted law of micropore nucleation and evolution during metal fatigue and thus can be regarded as a reasonable numerical prediction for the atomic-scale damage mechanism.

**Figure 30:**
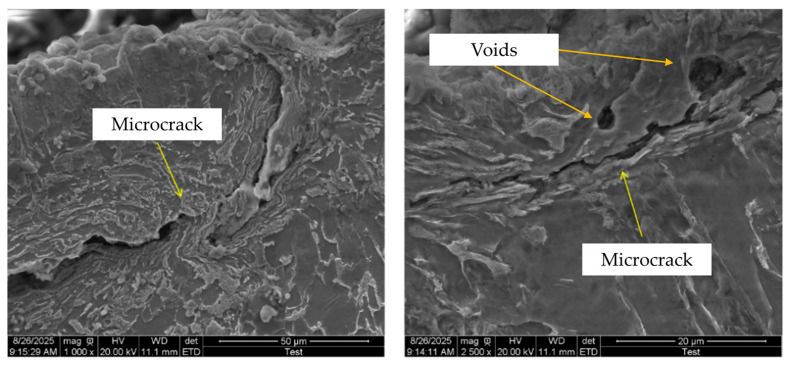
SEM image of the HAZ in AH36 welded specimens after fatigue testing.

The nucleation and evolution of voids observed in molecular dynamics (MD) simulations originate from the aggregation of atomic vacancies and local plastic deformation under stress concentration. These are natural numerical outcomes driven by physical mechanisms rather than numerical artifacts. Scanning electron microscopy (SEM) observations of the heat-affected zone (HAZ) in AH36 welded specimens after fatigue testing (see Figure 30) reveal the presence of microcrack and void defects in the HAZ, which indirectly corroborate the MD simulation results of the microscopic model.

However, in situ dynamic observation of nanoscale voids under fatigue cycling is extremely challenging, and direct experimental evidence corresponding to atomic simulations remains scarce. Nevertheless, the phenomena obtained from the microscopic model simulations are consistent with the well-recognized mechanism of micropore nucleation and evolution during metal fatigue and can thus serve as a reasonable prediction for the atomic-scale damage mechanism.

## 5. Conclusions

In this study, a multi-scale model was constructed using the molecular dynamics–finite element (MD-FEM) coupled multi-scale method, which realized the multi-scale information transfer and cyclic simulation of fatigue crack propagation in the welded heat-affected zone. Meanwhile, the MD-FEM multi-scale approach is a hybrid numerical method based on zonal coupling. For regions with small deformation and far away from defects, the finite element method is employed for a solution on the basis of the continuum theory, which can efficiently obtain the overall stress field and displacement field of the structure. For local regions with concentrated deformation and critical defects such as cracks, the molecular dynamics model is adopted for refined simulation to accurately capture the micro-deformation mechanisms of defect evolution and crack propagation. Benefiting from the core idea of such zonal coupling calculations, molecular dynamics only needs to focus on the microscopic core region near the crack tip, which is the critical space where micro-mechanisms including plastic deformation and void nucleation take place. Therefore, it is fully feasible that the molecular dynamics model in this study contains only approximately 96,500 atoms. Affected by the approximate treatment of the transfer mode of model boundary conditions, some calculation results present certain deviations in quantitative values. Nevertheless, the variation trends, physical mechanisms and core laws are consistent with the micro-mechanisms of metal fatigue damage accumulation and the fatigue crack propagation laws of AH36 steel, which does not affect the reliability of the law and mechanism analysis in this paper. This multi-scale simulation method can be applied to other welded zones, and the research and analysis of microscopic mechanisms can be carried out by establishing corresponding molecular dynamics microscopic models for other welded regions.

Based on the present multi-scale simulations, the following conclusions regarding the micro-mechanisms of fatigue crack propagation in the heat-affected zone of AH36 steel are drawn:

1. Crack propagation is influenced by the coupled effects of multiple factors. There is no monotonic relationship between the peak boundary strain and the crack propagation rate. When the peak strain varies, the propagation rate may show retardation due to factors such as crack morphology (blunting/sharpening) and propagation direction (e.g., longitudinal widening impeding forward propagation). This result is consistent with the experimental law of fatigue overload retardation in AH36 steel, and a comprehensive analysis combining multiple factors is necessary.
2. Crack tip passivation slows down the propagation velocity: During fatigue load cycles, the crack tip is passivated. This phenomenon changes the crack’s growth morphology (such as widening or turning) and directly leads to a decrease in the crack’s propagation velocity during subsequent cycle stages.
3. The crack tip stress field periodically induces voids: During fatigue crack propagation, voids nucleate in the regions of other-type atom aggregation identified by the CAN method. The differences in the number and size of voids reflect the degree of micro-damage accumulation to a certain extent. Plastic deformation at the crack tip gives rise to atomic lattice distortion and the aggregation of other-type atoms, and the structural disorder provides favorable conditions for void nucleation. The nucleation, aggregation and coalescence of voids further promote the formation and propagation of cracks. Meanwhile, some voids exhibit a healing phenomenon, which reduces the crack propagation rate to a certain extent. This may be one of the reasons for the relatively low crack propagation rate at the initial stage of crack nucleation.

## Figures and Tables

**Table 1:** Chemical composition range of AH36 steel specified in ASTM A131/A131M-19.

Category	Chemical Composition (Mass Fraction %)					
	C	Si	Mn	Cr	Ni	Cu
AH36	0.18	0.10–0.50	0.90–1.60	0.20	0.40	0.35

**Table 2:** The peak boundary strain, crack propagation length, crack propagation velocity and total crack propagation length at the corresponding cycle number during 600 cycles of loading.

Number of Load Cycles	Peak Boundary Strain	Crack Propagation Length (Å)	Crack Propagation Velocity (Å/cycle)	Total Crack Propagation Length (Å)
0–50	0.077	15	0.3	65
50–100	0.113	22	0.44	87
100–150	0.107	27	0.54	114
150–200	0.125	17	0.34	131
200–250	0.117	35	0.70	166
250–300	0.127	40	0.80	206
300–350	0.132	56	1.12	262
350–400	0.137	32	0.64	294
400–450	0.150	28	0.56	322
450–500	0.147	32	0.64	354
500–550	0.160	42	0.84	396
550–600	0.160	73	1.44	469

## Data Availability

The original contributions presented in this study are included in the article. Further inquiries can be directed to the corresponding author.
